# Characterization Methods for Nanoparticle–Skin Interactions: An Overview

**DOI:** 10.3390/life14050599

**Published:** 2024-05-08

**Authors:** Valentyn Dzyhovskyi, Arianna Romani, Walter Pula, Agnese Bondi, Francesca Ferrara, Elisabetta Melloni, Arianna Gonelli, Elena Pozza, Rebecca Voltan, Maddalena Sguizzato, Paola Secchiero, Elisabetta Esposito

**Affiliations:** 1 Department of Translational Medicine, University of Ferrara, 44121 Ferrara, Italy; dzyvnt@unife.it (V.D.); rmnrnn@unife.it (A.R.); elisabetta.melloni@unife.it (E.M.); elena.pozza@unife.it (E.P.); 2 Laboratorio per le Tecnologie delle Terapie Avanzate (LTTA) Centre, University of Ferrara, 44121 Ferrara, Italy; rebecca.voltan@unife.it; 3 Department of Chemical, Pharmaceutical and Agricultural Sciences, University of Ferrara, 44121 Ferrara, Italy; walter.pula@unife.it (W.P.); agnese.bondi@unife.it (A.B.); francesca.ferrara@unife.it (F.F.); maddalena.sguizzato@unife.it (M.S.); 4 Department of Environmental and Prevention Sciences, University of Ferrara, 44121 Ferrara, Italy; arianna.gonelli@unife.it

**Keywords:** nanoparticles, skin, transmission electron microscopy, fluorescence microscopy, confocal microscopy, hyperspectral microscopy

## Abstract

Research progresses have led to the development of different kinds of nanoplatforms to deliver drugs through different biological membranes. Particularly, nanocarriers represent a precious means to treat skin pathologies, due to their capability to solubilize lipophilic and hydrophilic drugs, to control their release, and to promote their permeation through the stratum corneum barrier. A crucial point in the development of nano-delivery systems relies on their characterization, as well as in the assessment of their interaction with tissues, in order to predict their fate under in vivo administration. The size of nanoparticles, their shape, and the type of matrix can influence their biodistribution inside the skin strata and their cellular uptake. In this respect, an overview of some characterization methods employed to investigate nanoparticles intended for topical administration is presented here, namely dynamic light scattering, zeta potential, scanning and transmission electron microscopy, X-ray diffraction, atomic force microscopy, Fourier transform infrared and Raman spectroscopy. In addition, the main fluorescence methods employed to detect the in vitro nanoparticles interaction with skin cell lines, such as fluorescence-activated cell sorting or confocal imaging, are described, considering different examples of applications. Finally, recent studies on the techniques employed to determine the nanoparticle presence in the skin by ex vivo and in vivo models are reported.

## 1. Introduction

Skin is the widest organ of our body acting like a barrier through exogenous trigger factors (e.g., UV light, pathogens, and mechanical factors). Skin physiologically renews more rapidly compared to most other tissues counteracting long-term detrimental effects of stressor exposure.

Indeed, the anatomical architecture based on tightly joined epidermal cells organized in multiple structural layers supports skin functions and, at the same time, represents a challenge for drug delivery [1]. The outer layer of the skin, the epidermis, is constituted mainly by keratinocytes that divide and differentiate from the inner basal membrane through the outer layer, forming the stratum corneum, where anucleate keratinocytes are called corneocytes [2]. In addition, the lipid matrix, mainly consisting of ceramides, fatty acids, and cholesterol organized in bilayers confers to the skin upper layer, is also a lipophilic character.

Due to its complex structure, the stratum corneum represents a limiting step for drug penetration and delivery to the epidermis and dermis [3]. Given its protective function against external insults and environmental stressors, the skin is exposed to several risks spanning from infections to physical and mechanical injuries. Apart from conventional topical semisolid formulations, such as gels, creams, and ointments, research efforts have led to the development of nanoplatforms for cutaneous administration. For instance, some inorganic nanoparticles (NPs), such as nano-sized silver, titanium oxide, and zinc oxide, can be employed as antimicrobial or photoprotective agents without penetrating the skin.

Instead, for the treatment of many skin pathologies, several nanocarriers delivering bioactive molecules (of either synthetic or natural origin) have been considered, such as polymeric NPs, solid lipid nanoparticles (SLNs), cubosomes, liposomes, transfersomes, ethosomes, and transethosomes [4,5]. 

Notably, different nanosystems have been in vitro or in vivo investigated for the treatment of skin disorders due to aging, inflammation, wounds and bedsores, fungal infections or psoriasis, melanoma, and other skin cancers [6,7,8]. Additionally, topical and transdermal applications can be used as non-invasive skin disease treatments that are often associated with better patient compliance [3]. Indeed, the topical application of nanosystems allows a deep interaction with the lipid matrix and cutaneous strata, promoting a controlled release of the loaded molecules. Some characteristics of nanocarriers, such as physicochemical aspects (size, shape, and surface charge), the source of the matrix used, and environmental factors (temperature, osmolarity, and pH), influence their penetration and uptake [9].

In this respect, the characterization of NPs plays an important role in the development of efficacious delivery systems for skin disease treatment. Skin penetration tests are necessary to confirm the performance of topical or transdermal products. To assess the therapeutic potential of nanocarriers, skin penetration evaluation can be employed to detect their presence in the different skin layers [10]. In this regard, a review article by Raszewska-Famielec and Flieger describes the influence of nanocarrier physical–chemical properties and the experimental models employed to detect skin penetration [10].

Due to the encouraging preliminary results of nanocarrier-based drug delivery in the treatment of skin diseases, the methods needed for nanocarrier characterization and analysis of their interaction with skin are constantly increasing. Based on that, this review aims to update some of the main approaches useful to characterize nanocarriers and their interaction with the skin, mainly focusing on microscopy methods. 

## 2. Nanocarriers for Skin Applications 

The use of nanocarriers has emerged as a promising strategy to treat skin disorders and pathology, possibly passing the epidermal barrier while minimizing tissue damage and facilitating efficient drug penetration. Table 1 delineates various prevalent drug nanocarriers, encompassing polymeric NPs, lipid NPs (such as liposomes, ethosomes, SLNs, and nanostructured lipid carriers), nanoemulsions, dendrimers, micelles, and inorganic NPs tailored for transdermal delivery.

In the category of NPs, polymeric nanocapsules and nanospheres represent promising tools to increase the cutaneous permeation of pharmaceuticals and bioactive agents. Nanocapsules, characterized by their oil content, exhibit a vesicular architecture, while nanospheres, lacking oil, present a matrix-like arrangement of polymeric chains [70]. These nanoparticulate systems possess the ability to encapsulate, disperse, solubilize, or adsorb drugs, thereby facilitating their transdermal delivery. Their use in dermatological and cosmetic formulations demonstrates significant potential for enhancing drug efficacy while mitigating systemic absorption, with a primary focus on extending dermal permeation and the transport of bioactive compounds [71].

In recent years, lipid nanocarriers have become increasingly significant due to their numerous advantages in treating skin diseases [72]. They enable the delivery of drugs to deeper skin layers, maintaining and possibly increasing skin hydration due to the structural similarity between the nanocarrier lipid matrix and that of the stratum corneum [73]. Lipid nanocarriers encompass diverse drug delivery systems, including phospholipid nanovesicles organized in bilayered lamellar structures, such as liposomes. Other examples are SLNs and nanostructured lipid carriers, which are especially suitable for lipophilic drug loading [74]. Promising results in treating skin pathologies have been reached using ethosomes, second-generation liposomes made of an aqueous dispersion of phosphatidylcholine in ethanol [75]. Ethanol gives them a softer and more malleable nature concerning liposomes and fluidizes the lipid bilayer, enhancing transdermal permeation [73]. Several authors have demonstrated that the formulations of drugs and ethosomes display high efficiency in transdermal delivery, showing improved permeation through the skin without irritation for controls and maintaining the properties of the delivered drug to the target site [75,76,77].

Nanoemulsions are advanced drug delivery systems characterized by a particular composition consisting of an aqueous phase, an oil phase surfactants, and co-surfactants that permit homogeneity and thermodynamic stability. These formulations typically feature droplet sizes ranging from 20 to 200 nm, depending on the composition and the homogenization technique. Their potential lies in their ability to augment the solubility and bioavailability of bioactive compounds and pharmaceuticals, thereby enhancing therapeutic outcomes [78,79].

Micelles, particularly polymeric micelles, present an intriguing avenue for skin drug delivery due to their capacity to enhance drug solubility and stability, potentially improving therapeutic outcomes. Studies have shown that polymeric micelles, with particle sizes around 200 nm, can penetrate the skin, reaching keratinocytes and fibroblasts. They may even accumulate in hair follicles, suggesting their potential for targeted drug delivery to specific skin regions [80,81].

Dendrimers, characterized by their well-defined, hyperbranched structure, offer unique opportunities for tailored drug delivery and targeting in the context of skin applications. Their precise manipulable size, shape, and functional groups make them promising candidates for enhancing skin delivery, with potential benefits including reproducible pharmacokinetic behavior and the ability to encapsulate drugs within their cavities or to conjugate them to surface groups [82,83].

Inorganic NPs, typically ranging in size from 1 to 100 nm, exhibit distinct physical, chemical, and biological attributes due to their high surface-to-volume ratio and quantum effects. For instance, gold NPs, silica NPs, silver NPs, quantum dots, silica, titanium dioxide, and zinc oxide particles are prominent types demonstrating significant functionalities relevant to skin-related applications [84].

Notably, in the development of NP formulations aimed at skin application, researchers should firstly characterize the produced nanocarrier to investigate its main physicochemical features, such as size, shape, and inner structure. After verifying the appropriateness of the NPs for the route of administration, researchers should investigate its effect on the skin by in vitro, ex vivo, or in vivo approaches. 

## 3. Nanocarrier Physicochemical Properties and Characterization Techniques 

It is noteworthy to underline that all types of NPs are characterized by crucial features that strongly influence their stability, as well as their fate upon administration. In this respect, comprehensive investigations are required to elucidate the physical attributes of NPs. Indeed, the interactions between biological barriers and NPs depend on the physicochemical properties of the nanocarriers [85,86].

Particularly, the main physical and chemical NP characteristics, such as size distribution, shape, inner structure, and surface charge, should be accurately evaluated in the development of drug formulations designed for skin applications. 

### 3.1. NP Size Distribution

Particle size is a crucial factor affecting skin penetration; indeed, since the nanocarrier size distribution can widely affect the membrane permeability, it is one of the first parameters to be accurately evaluated. Different methods are used to study NP dimensions, including dynamic light scattering.

Dynamic light scattering (DLS), also known as photon correlation spectroscopy (PCS), stands as a cornerstone technique extensively employed for the measurement of particle size and for conducting size distribution studies, particularly in colloids and polymers dispersed in liquid media, with a measurement range typically spanning from 3 nm to 3 μm [87]. The fundamental principle of DLS is based on the Brownian diffusion of spherical particles within a solution. As these particles undergo Brownian motion, they induce fluctuations in the intensity of scattered light when subjected to a beam of laser light emitted into the NP solution. The hydrodynamic radii of nanoparticles, as determined by DLS, provide valuable insights into their behavior in dispersed systems [88]. One of the critical parameters evaluated through DLS is the polydispersity index (PDI), which indicates the uniformity of particle size distribution within a sample. A PDI value ranging from 0.1 to 0.25 signifies a narrow size distribution, while values exceeding 0.5 indicate a broader distribution [87].

DLS presents several advantages in an NP analysis since (i) it offers rapid measurement capabilities, enabling quick particle size and size distribution assessments with a minimal sample preparation, and (ii) it is non-destructive, allowing for repeated measurements without compromising the sample’s integrity. In addition, DLS allows us to perform measurements under physiological conditions, mimicking in vivo behavior and providing insights into the hydrodynamic sizes of NPs in biological fluids [89]. DLS finds extensive applications in various fields, including the evaluation of the formulation stability over time and temperature variations, identification of aggregates in different formulations, and a rapid determination of particle size in monodisperse samples [88].

Moreover, DLS is versatile and applicable across various scientific disciplines, facilitating studies in colloidal chemistry, biochemistry, biophysics, molecular biology, and food technology [90].

Despite its utility, DLS/PCS does have certain limitations. Firstly, it may not be possible to determine particle size for samples that absorb light at the same wavelength as the laser utilized in the equipment. Additionally, DLS measures the equivalent sphere hydrodynamic diameter, which lacks information on NP shape and potentially underestimates particle size in suspension [87]. Moreover, larger particles scatter laser radiation more efficiently than smaller ones, posing challenges in accurately measuring particles in the presence of dust or agglomerates [91].

### 3.2. NP Shape

The shape of NPs can influence their stability in terms of spontaneous aggregation or agglomeration, as well as biological activities such as cell proliferation, differentiation, and metabolism [91]. 

Notably, the NP shape affects the cellular uptake mechanisms; indeed, nonspherical particles, such as discoidal or rod-shaped, often exhibit better adhesion compared to spherical particles [92]. Arno and colleagues demonstrated that, in the case of gel nanocomposites, platelet-shaped poly(*L*-lactide)-based nanoparticles significantly improved cell adhesion with respect to spherical or cylindrical micelles [90]. Remarkably, in the case of peculiar shapes, such as spiky nanoprisms or branched nanostructures, the NP morphology can cause cytotoxic effects due to cell death [91]. 

In this respect transmission electron microscopy (TEM) stands as a pivotal tool to study NP shape, alongside atomic force microscopy (AFM) and scanning electron microscopy (SEM). Since TEM offers detailed insights into the morphology and structural characteristics of drug nanocarriers, it becomes valuable both for the formulation development and for in vitro/in vivo investigations [93].

TEM enables imaging of the specimen structure by transmitting a high-voltage electron beam (80–300 kV) through a sample. Various sources, including thermionic sources and field-emission sources, generate standard electron beams, which are accelerated by an anode at voltages ranging from 40 to 400 kV. The accelerated beam transmits through electrostatic and electromagnetic lenses, focusing on the specimen, and the resulting image is produced by collecting and magnifying the electrons with objective lenses. Advanced technologies such as new detectors and high-tech CCD cameras enhance image quality and resolution, visualizing details of NPs crossed by the electron beam [94,95,96]. TEM offers several advantages in characterizing liquid dispersions of nanoparticulate systems, providing detailed information on particle morphology, as well as on size, crystallinity, and interparticle interactions at high resolution (~0.07 nm). Moreover, TEM can be a potent complementary instrument to conventional particle sizing techniques, offering sensitivity and selectivity in determining interactions during nanoparticle fabrication [97]. Despite its advantages, TEM has limitations since the stained technique enables the “negative” color of the samples, resulting in grayscale, two-dimensional images [98,99]. 

Conversely, a better resolution of NP morphology can be obtained by cryogenic electron transmission electron microscopy (cryo-TEM). The method is based on the preparation of specimens by the “thin film” technique that allows us to obtain vitrified samples by rapid immersion into liquid ethane or propane cooled to approximately −170 °C. The visualization of unstained, vitrified specimens sheds light on the supramolecular organization of nanoparticulate structures, providing high-quality images of NPs, revealing the inner organization of the particulate or vesicular disperse phases [100]. 

**Figure 1:**
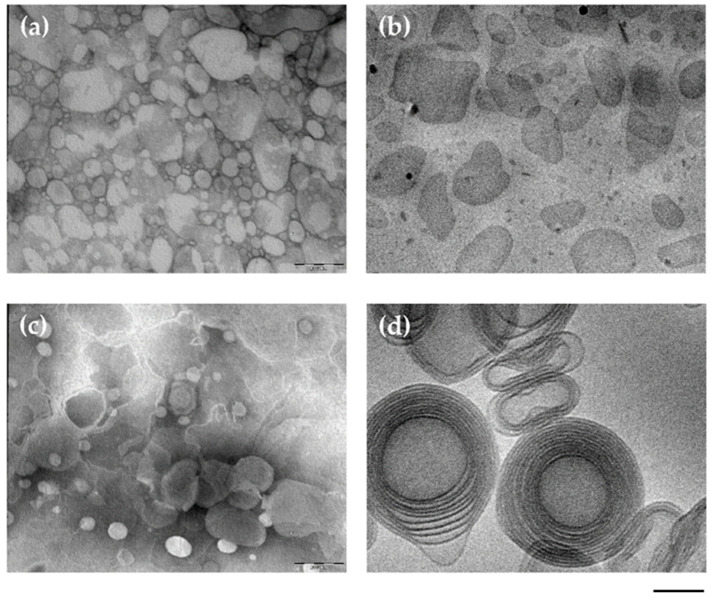
Caffeic acid-containing solid lipid nanoparticles (**a**,**b**) and caffeic acid-containing ethosomes (**c**,**d**) obtained by transmission (**a**,**c**) or cryogenic transmission (**b**,**d**) electron microscopy. The bar corresponds to 150 nm in panels (**a**–**c**) and 50 nm in panel (**d**) [5].

The morphology of different caffeic acid-loaded lipid nanoplatforms was compared by TEM and cryo-TEM in a study by Hallan et al.; particularly, SLN and ethosomes were evaluated. Figure 1 shows microphotographs of caffeic acid-loaded SLN (a,b) and caffeic acid-loaded ethosomes (c,d) obtained by TEM (a,c) and cryo-TEM (b,d). The differences between the two types of nanoplatforms are appreciable. Indeed, SLNs show an irregular and flat shape both by TEM (a) and cryo-TEM (c). Remarkably, in the case of ethosomes, cryo-TEM revealed the typical fingerprint structure of multilamellar spherical and ovoidal vesicles, suggesting their similarity with the biological membranes [5]. 

AFM imaging is another useful technique to comprehensively characterize micro/nanocarriers due to its inherent advantages. Indeed, AFM provides high-resolution imaging capabilities, exploring the intricate 3D structures at the nano-scale level. By AFM the morphology and surface attributes of NPs can be detected, elucidating structural organization and their associated applications [96]. Particularly, by AFM the shape of spherical inorganic NPs can be well investigated. In addition, the AFM technique is employed to study the mechanical properties of NPs encompassing parameters like elasticity, stiffness, and adhesion. Insights into the mechanical behavior of the carriers across diverse environments, including liquid settings, are gathered by carefully examining interaction forces between the AFM tip and the specimen [101]. In addition, AFM facilitates in-depth investigations into the interaction dynamics between nanocarriers and biological entities such as cells and tissues. Indeed, functionalizing the AFM tip with specific ligands or biomolecules can thoroughly assess the binding affinity and specificity of nanocarriers toward target cells or tissues [102]. Critical insights into the kinetics and mechanical foundations of drug release and cellular uptake processes can be obtained by monitoring morphological and mechanical changes in nanocarriers over time scales [103]. Anyway, the AFM technique presents several noteworthy limitations. Indeed, the necessity of sample adsorption onto the substrate surface during AFM imaging can induce alterations in the sample’s native morphology and dimensions, potentially compromising the accuracy of the acquired data. Moreover, interactions between the AFM tip and the sample surface may result in structural damage, particularly under conditions of excessive pressure or prolonged scanning durations, introducing the possibility of misinterpretation of experimental outcomes [96].

The shape of solid-state dry nanocarriers can be investigated by SEM. By this technique an accelerated beam of electrons is generated in vacuum and applied on solid-state specimens. The scanning of the electron beam on the NP surface gives diverse signals, including the reflection of high-energy electrons, radiation of secondary electrons, and emission of X-rays, which can be discreetly detected and analyzed by specific detectors, resulting in detailed imaging of nanocarrier structures [96]. The benefits of SEM in characterizing nanocarriers lie in its high resolution and accuracy, allowing for the assessment of structural and morphological features critical for understanding delivery system functionality. SEM’s ability to achieve high resolutions down to a few nanometers and its flexible field of view allows the studying of NPs ranging in size from 0.1 to 1000 μm. Despite these advantages, SEM has some limitations, including its inability to characterize the internal structure of specimens with the same resolution as techniques like TEM. Moreover, the need for appropriate specimen preparation and vacuum operation can result in artifacts and morphological variations from the original state of the specimen [88]. Innovative SEM techniques such as low-vacuum SEM or environmental SEM have emerged to address these limitations, eliminating the need for conventional specimen preparation and enabling visualization of specimens in their native state under ambient conditions [96,104]. Remarkably, SEM can be employed as a precious tool to detect the skin absorption of inorganic nanoparticles leading to toxicity. Particularly some SEM studies demonstrated the presence of Au and Ag NPs isolated or as clusters on skin samples [105].

### 3.3. NP Inner Structural Organization

The inner architecture of NPs is dictated by the materials employed for their preparation, as well as from preparation procedures. Some biomaterials, such as lipids, employed to build biocompatible nanocarriers, can generate different nanostructures as a function of their concentration, amount of water, and temperatures. For instance, glyceryl mono-oleate in water can form different liquid crystal structures, spanning from micelles to lamellar, hexagonal or cubic phases, simply changing the preparation modalities and percentage of components [73]. The different crystalline order of NPs can affect the release modalities in the encapsulated drugs, the biodegradation, the cellular uptake, and thus, the final therapeutic effect. 

In this respect, X-ray scattering techniques are precious means for characterizing nanocarriers, providing valuable insights into their structural properties and organization at different length scales. Wide-angle X-ray scattering explores angles greater than 10°, enabling detailed examination of atomic-scale structures ranging from approximately 1 to 10 Å. This technique, also known as wide X-ray diffraction, generates Bragg peaks as a function of the scattering angle, offering information about the crystalline nature, crystallinity degree, and orientation of mesoporous materials and crystalline lipid nanoparticles [106,107]. Furthermore, small-angle X-ray scattering (SAXS) detects radiation at angles between 0.1° and 10°, providing information on structures within a range of approximately 10 to 1,000 Å. SAXS is sensitive to electron density differences and can elucidate the shapes, sizes, and spatial distribution of dispersed particles, including macromolecules, polymers, micelles, and nanoparticles. Additionally, SAXS can explore interactions within the system and average correlation distances, making it a valuable tool for characterizing nanocarriers and their assemblies [88]. Moreover small-angle neutron scattering and small-angle light scattering are other techniques employed for small-angle scattering, giving valuable information about the structures of nanocarriers and their interactions within the sample environment [108].

X-ray diffraction was employed to investigate the inner structural organization of glyceryl mono-oleate aqueous dispersions as curcumin cutaneous delivery systems. It was found that the use of different surfactants led to various crystalline structures. Indeed, Na cholate or Na caseinate formed only vesicular systems (diffuse scattering by X-ray) while the presence of curcumin or poloxamer led to the formation of ordered cubic phases. The mixing of Na cholate and Na caseinate with glyceryl mono-oleate resulted in different structures such as hexagonal phases, while bentonite associated with Na cholate formed both cubic and hexagonal phases [109].

Furthermore, to study the complex interactions between NP components, bioactive–NP, bioactive–medium (biological), and NP–medium, the Fourier transform infrared (FTIR) analysis can be used. By exploiting the infrared (IR) absorption bands of various chemical species as manifestations of their structural vibrations, FTIR methodology enables scrupulous investigations into the molecular composition and interactions inherent to nanocarrier systems. FTIR is employed in the analysis of nanomaterials, particularly for detecting functional groups present on their surfaces. This technique enables the assessment of surface-modified nanomaterials aimed at enhancing their affinity, reactivity, or compatibility for various applications. By identifying the functional groups through FTIR analysis, the most suitable surface modification strategies can be determined. Moreover, FTIR facilitates the characterization of surface modifications by detecting new functional groups resulting from successful reactions [110,111]. Recent advances in FTIR spectroscopy, particularly the integration of focal plane array detectors, have introduced FTIR imaging. This advanced technique gains insight into the three-dimensional spatial distribution of specific chemical constituents within nanocarriers, investigating their structural details [111]. The characteristic bands obtained by FTIR spectroscopy represent “fingerprints”, shedding light on the conjugation between the materials employed for NP preparation and the association with encapsulated drugs. In a recent study by Pasalic et al., a FTIR spectroscopy was employed to characterize asymmetric lipid membranes of liposomes, giving information on the influence of the preparation process on mechanical and structural properties in asymmetric large unilamellar vesicles [112].

Raman spectroscopy is a valuable analytical tool for studying nanocarriers. Indeed, lipids, essential constituents in many colloidal systems of pharmaceutical relevance, exhibit Raman spectra sensitivity to conformational, packing, and dynamical changes involving hydrocarbon chains. This sensitivity enables the evaluation of hydrocarbon chain states, including the population of trans and gauche conformers, packing behavior, and phase transitions [113,114]. Raman scattering processes provide insights into molecular vibrations within samples, with the energy difference between incident and scattered photons corresponding to the energy required to excite specific molecular vibrations. Consequently, each molecule produces a characteristic Raman spectrum, making it a useful tool for molecular analysis [114]. Compared to other techniques like FTIR, Raman spectroscopy offers distinct advantages being not susceptible to interference from aqueous environments, resulting in straightforward sample preparation and handling. Extensions of excitation wavelengths to the near-infrared region and the development of surface-enhanced Raman scattering have addressed some AFM limitations, due to low sensitivity and sub-sampling issues, leading to an upsurge in its applications for pharmaceutical analysis, including the evaluation of liposomes employed both as diagnostics and therapeutics [115,116].

### 3.4. NP Surface Charge

Surface charge significantly influences the behavior of NPs, impacting their stability, as well as interactions with cells and proteins. The NP surface charge can be measured by Zeta Potential (ZP), also known as the electrokinetic potential [117]. ZP measurements involve the application of an electric field, and the electrophoretic mobility of particles is determined through electrophoretic light scattering. This technique involves the scattering of incident laser light by mobile particles during electrophoresis [118]. Various factors influence the ZP of NPs in colloidal systems, playing a crucial role in determining their stability and surface properties. The pH value exerts a significant impact on ZP in aqueous dispersions. Generally, ZP becomes more positive with an acidic pH and more negative with a basic pH. The identification of the isoelectric point, where ZP equals zero, through titration curves is indicative of colloidal stability [119,120].

Regarding colloid stability, ZP data categorize NP dispersions into stability classes. However, it is essential to note that ZP alone does not provide complete information on colloid stability, as it solely reflects electrostatic repulsive forces [121]. ZP is a valuable tool for assessing the surface charge of NPs, with positive or negative values indicating respective charges. However, caution is necessary when associating ZP with surface charge, as it does not measure charge density directly. Confirmatory techniques, such as titration, are essential for accurately determining charge density [122]. The skin-negative charge of corneocyte components should be considered concerning nanocarrier skin interaction. Some studies demonstrated that positively charged NPs can better penetrate the skin due to electrostatic attraction, while negatively charged NPs lead to aggregation on the skin surface [123,124].

Specifically, in inflamed skin, negatively charged NPs tend to accumulate more than their cationic counterparts. Furthermore, both negatively and positively charged particles showed greater effectiveness in treating experimental dermatitis compared to neutral carriers [125].

## 4. Nanoparticle Interaction with Skin and Cellular Uptake 

**Figure 2:**
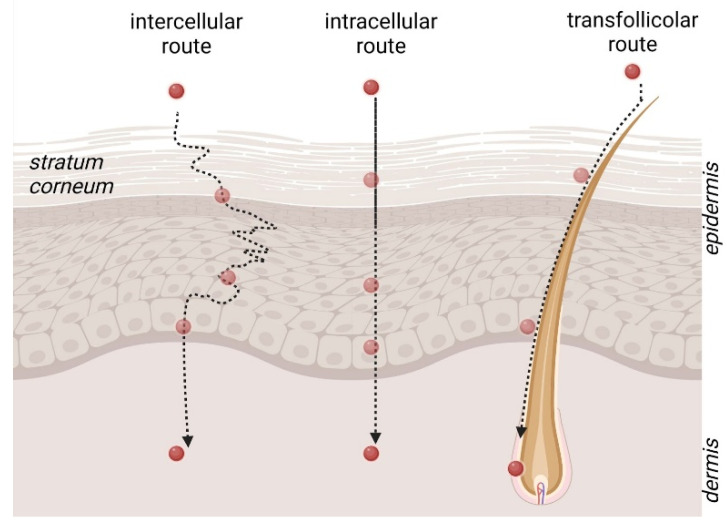
The different ways nanocarriers can penetrate through the skin. Original figure created with BioRender.com.

In order to exert their therapeutic function, nanocarriers should pass through the skin, deliver the loaded drug, and undergo degradation without skin irritation or toxic effects. When penetrating the skin, NPs encounter potential challenges. Notably, the complex structure of stratum corneum represents the first main barrier for NP passage. The way different NP matrices could account for different penetration mechanisms is still a debate point. As a general rule, as represented in Figure 2, in the case of intact, undamaged skin, nanocarriers can penetrate crossing the stratum corneum lipid matrix in three different ways: by intracellular route, by intercellular route, or by transfollicular route, i.e., through the sweat gland pores, sebaceous glands, or hair follicles [10]. By the intracellular pathway, NPs should cross the corneocytes, passing inside the cells, thus in hydrophilic regions or in the lipophilic regions of the extracellular matrix. On the other hand, many authors demonstrated the nanocarrier deposition in skin appendages such as hair follicles, suggesting the possibility to prolong drug release by nanocarrier accumulation. 

NPs interaction with cells is a crucial point influencing the cellular responses to loaded therapeutics. Indeed, as a function of the above-mentioned physical-chemical parameters, the nanocarrier’s role is the promotion of drug passage and release through the epidermis/dermis, reaching, in some cases, a systemic effect due to transdermal delivery. Stratum corneum and skin-tight junction limit the NP passage; however, some NPs are facilitated in the delivery thanks to smaller dimensions compared to the cell gaps. NPs with diameters < 10 nm or a molecular weight < 600 Da can reach the systemic circulation, passively crossing the skin barriers [10]. Conversely, some authors detected the presence of NPs and nanovesicles of 200–300 nm in the upper skin layers [126]. Penetration also becomes straightforward in damaged skin, like in psoriasis and atopic dermatitis, where uncontrolled proliferation leads to a loss of epidermis barrier integrity [127,128]. 

In the case of deeper penetration, NPs interact with the cell membrane, a phospholipid bilayer with hydrophobic heads orientated through the extracellular space and the cytosol and hydrophilic tails in the inner portion, an amphiphilic structure with selective proprieties that ensure cellular homeostasis [9].

As mentioned above, NPs interactions are highly dependent on the chemical composition, shape, size, functional groups on the NP surface, stiffness, and hydrophobicity/hydrophilicity that can enhance or reduce the NP adhesion and uptake. Uptake is also highly dependent on the type of target cells, for instance, internalization became easy in specialized cells for particle internalization like skin immune cells [128].

The NPs penetration in the cells takes place mainly by endocytosis (clathrin- and caveolae-mediated endocytosis) or phagocytosis [3,129].

NPs between 20 and 100 nm can be internalized by caveolin-mediated endocytosis, whereas NPs in the range of 100 to 350 nm mainly follow the clathrin root [123]. In both cases, firstly, NPs interact with the cell membrane through receptor-mediated interactions or non-specific interaction, such as hydrophobic or electrostatic interactions; afterwards, the membrane invaginates to form an endocytic vesicle that is internalized [130]. Clathrin-mediated vesicles inside the cells fuse with lysosomes exposing the internalized NPs to an acidic and enzymatic-rich environment that might compromise the pharmacological activity of drugs loaded into NPs. Differently, caveolin-mediated vesicles form cytosolic caveolar vesicles that are generally transported to the Golgi, bypassing lysosome degradation pathways [129].

**Figure 3:**
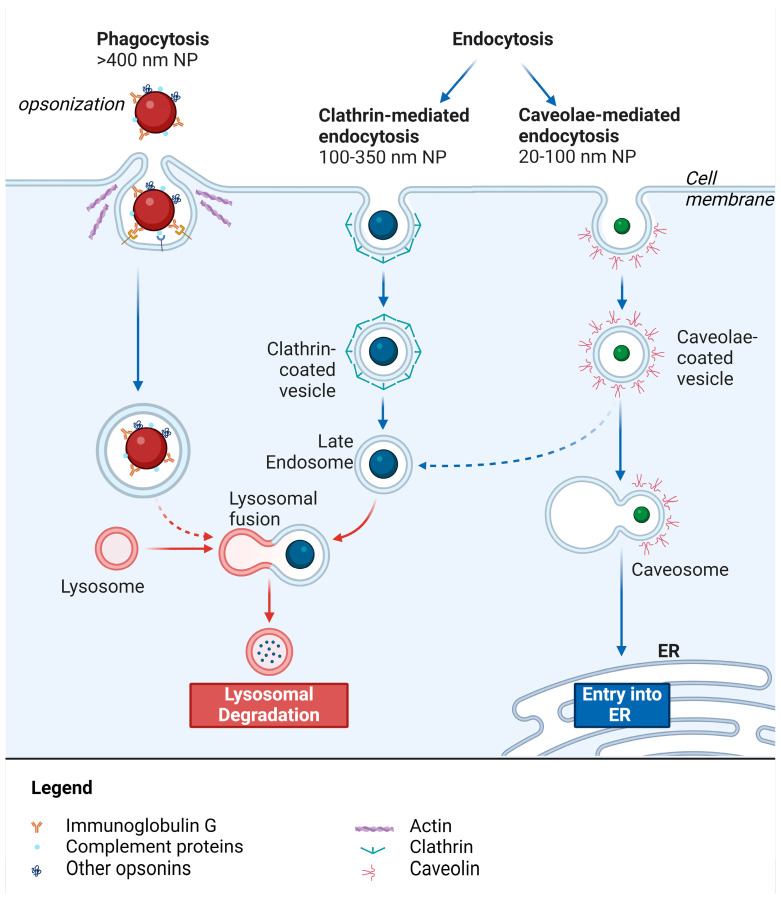
The different nanocarrier uptake mechanisms. Original figure created with BioRender.com.

Large NPs (>400 nm) are uptaken through phagocytosis, which serves as main penetration route for specialized cells like macrophages or dendritic cells, while non-specialized phagocytes like fibroblasts can exert phagocytic activity but with marginal importance. The phagocytic process starts with opsonization, a process of association of extracellular matrix proteins with NPs. An opsonized NP is recognized and binds to specific receptors that trigger cytoskeleton modification for particle engulfment and internalization [9,123] (Figure 3).

An alternative mechanism of cellular entry for larger NPs includes macropinocytosis, a process that forms large vesicles, without implication of receptors, that physiologically is used for antigen presentation and is exploited for viruses and bacteria entry.

Other penetration methods such as passive diffusion or hole formation were reported, but to date, endocytosis represents the major uptake mechanism [9].

To investigate NPs uptake, and intracellular localization, imaging techniques like electron microscopy and fluorescence microscopy, implemented with 3D imaging protocols represent new opportunities for understanding nanomaterial interaction with tissues and cells [131,132,133]. 

## 5. Methods to Characterize Nanoparticle–Skin Interactions 

A synergy of different characterization techniques is pivotal to elucidate NPs behavior upon skin application and their consequential effects on the biological system [134,135]. Different investigative methods are required to identify NPs interaction with skin strata and cells. NP penetration and internalization capability needs to be standardized for a better understanding of the NP fate and data reproducibility. 

In vitro, primary or immortalized fibroblasts, keratinocytes, and melanocytes are the main type of cells used for preliminary investigations of NPs by electron microscopy techniques like TEM or SEM, or by fluorescence-based methods such as confocal imaging (Table 2).

Electron microscope techniques were mainly used to investigate the distribution of NPs inside the tissues of the cell compartments [144]. TEM resulted in a powerful tool to analyze the NP interactions with subcellular cell structures; conversely, SEM mainly focused on the cell surface interactions [144]. For instance, ethosome and transethosome vesicles were identified by TEM, inside the cytoplasm of the human keratinocytes cell line (HaCaT) and the primary dermal fibroblasts from a skin biopsy. They penetrated into the cells conserving their structure and without altering the viability of the cells as reported by organelles morphology and according to cytotoxicity studies [73,136,137].

Fluorescence-based methods were employed to easily track fluorescent NP interacting with cells or tissues. Fluorescence was based on the excitation of a fluorophore by light, which was partially absorbed and, in part, emitted as light with a longer wavelength compared to the one absorbed [145]. 

To measure the scattering and fluorescence of single particles, or cells in the stream, a flow cytometer was employed. The flow cytometry analyzed single cells suspended in a buffer solution flowing through a capillary. The method allowed the sorting of cells based on the fluorescence pattern, identifying specific morphologic characteristics of cells. For each cell, a single or multiple laser light generated a visible light scatter and revealed fluorescent signals collected by the detector [146]. By fluorescence-activated cell sorting (FACS), fluorescence-labeled NP interaction with single cells were visualized in a dynamic live investigation with high consistency data based on the high number of cells analyzed. HyeRim and colleagues quantified the level of fluorescein 5(6)-isothiocyanate-labeled silica NP uptake in some cell lines from different organs that can be primarily exposed to NPs, such as the skin. A flow cytometry-based assay was employed to investigate NP uptake, measuring the percentage of cells containing fluorescent NPs. Since the method did not allow quantifying the NPs inside the cell, the authors managed to develop a strategy based on multiple fluorescence channels that allowed them to measure the fluorescence intensity of a population of cells treated with NPs, providing information about the amount of NPs in the cells. In addition, the method allowed the correlation between the NP uptake and the cellular status such as the cell cycle, oxidative stress, and genotoxicity [138]. 

In order to investigate fluorescent-NP interactions with cells, also taking into account cell morphology, fluorescent microscopy was a more suitable approach. Using different color staining for NP and cell structures helped in visualizing the interaction of nanocarriers with intracellular organelles. Costanzo and colleagues, for instance, reported green fluorescence ethosomes and transethosomes localization inside the cytoplasms stained in red (trypan blue) and nuclei in blue (Hoechst 33342) [137].

Confocal laser scanning microscopy (CLSM), also known as confocal microscopy, provided the visualization of intracellular cell structures based on z-stack step scanning both from thin and thick samples [128,147]. Laser light was focused through a pinhole and collected in a “focus” plane that was moved over the sample to obtain the final (volumetric) image. In vitro and ex vivo investigation were associated with fluorescent staining (fluorescent confocal microscopy) for monitoring the fast dynamics in living cells or in fixed samples [147,148]. Rancan and colleagues reported by CLSM the internalization of FITC-silica NP in HaCaT cells with enhancing uptake mediated by positive charge on the NP surface [128]. Differently, Lee and colleagues, by confocal microscopy, demonstrated the cytotoxicity of ZnO NP with concentrations higher than 10 µg/mL in HaCaT cells reported changes in nucleus shape from uniform to vacuous [140]. Although confocal microscopes are ready to use and broadly available, for the best images, several parameters must be taken into account like an accurate choice of objectives, fluorophores, mounting medium, and optical components [148].

Another technique that can be applied both in vitro as well as ex vivo is Raman microscopy, a label-free technique that investigates the sample chemical composition with resolution from micron to submicron, depending on the weight length of the laser source. The high resolution enabled us to gain information about subcellular compartments, and combining with specific scattering of NPs, became a valuable tool to investigate NP–cell interaction [141]. Notably, silver NPs were identified by Raman microscopy in vitro into the cytosol of HaCaT and mouse embryonic fibroblasts [141,142].

Remarkably, hyperspectral light microscopy (HLM) is an innovative technique used for the analysis of NPs, leveraging spectral data acquisition alongside optical microscopy methods such as fluorescence, brightfield, or darkfield microscopy. This combination allows for the extraction of additional valuable information about the sample under examination. Hyperspectral imaging sensors are capable of capturing UV/Vis or infrared spectra from closely located different areas, organizing the obtained data as cuboids where the x and y dimensions represent spatial information, while the z dimension covers a specific wavelength range. Each pixel’s spectrum enables reliable identification of recorded objects, facilitating precise analysis and mapping [149]. Microscopes utilizing spectral identification of objects demonstrated the ability to detect and track NPs in vitro and in vivo, even in complex biological samples. This capability allowed investigating the potential biological effects and health implications of NP exposure, which are increasingly prevalent in various industrial processes and commercial products [150].

Roth et al. demonstrated the application of enhanced dark-field microscopy, coupled with hyperspectral imaging, as a promising avenue for identifying and characterizing metal oxide NPs in histological samples. This method facilitates efficient and cost-effective NP characterization, enabling high-contrast imaging and precise material identification at the nanoscale. By analyzing positive control samples to establish reference spectra and incorporating negative controls to enhance specificity, this approach enabled accurate mapping of NP distribution within tissue samples [150].

Beyond in vitro information on NP interaction with different skin cells, a more accurate view of drug-loaded NP fate can be achieved by studies on ex vivo tissues or directly from in vivo applications. Table 3 summarizes some studies devoted to understanding the interaction of NPs with animal or human skin. Particularly, both reconstituted human skin, as well as skin explants, were employed. Different kinds of microscopy techniques are useful, including TEM, fluorescence, and confocal microscopy, as well as Raman and hyperspectral microscopy.

**Figure 4:**
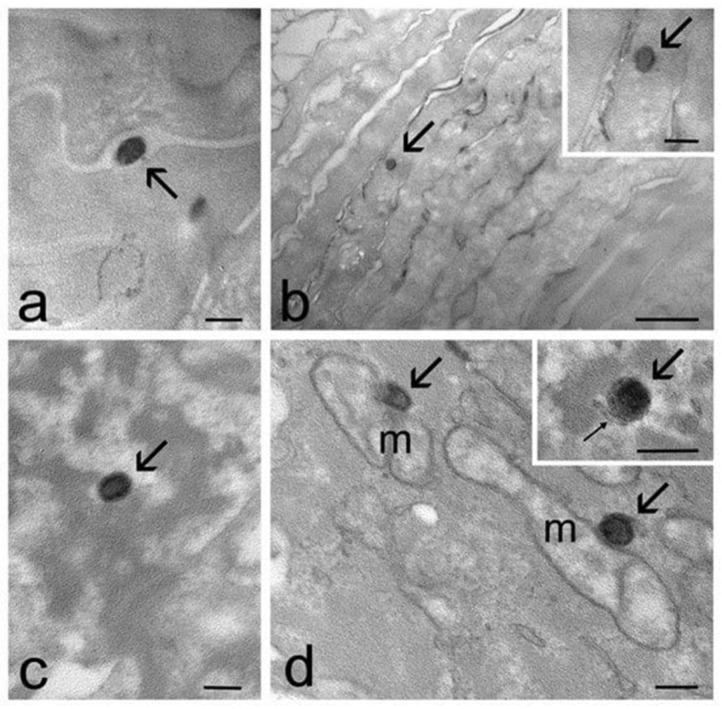
TEM micrographs of ethosome in the skin. (**a**) An ethosome (arrow) occurs in the intracellular space of the stratum corneum. (**b**) An ethosome (arrows) has been internalized in a corneocyte. In the high-magnification micrograph, the dark rim and the weakly electron-dense core of the ethosome (arrow) are clearly visible. (**c**) An ethosome (arrow) in the cytoplasm of a keratinocyte belonging to the stratum granulosum. (**d**) Ethosomes (arrows) make contact with mitochondria (m) and smooth endoplasmic reticulum cisternae (thin arrow in the inset) [151].

By TEM, nanocarriers like ethosomes and transethosomes were demonstrated to penetrate through epidermis strata of reconstituted human epidermis or skin explants overtaking the stratum corneum [136,151]. In particular, ethosome vesicles were ex vivo administered on human skin explants with the aim to gain information on their fate inside the epithelial strata. A bioreactor under fluid dynamic conditions associated with explanted human skin was employed to mimic the physiological state, preserving the functional structure. After incubation with ethosomes for different time periods (1–24 h), the skin was fixed and analyzed by TEM, taking untreated skin samples as the control. As reported in Figure 4, TEM was able to clearly identify the roundish, strongly electron-dense structure of ethosomes, showing a darker rim attributed to the double layer of phosphatidylcholine lamellar structure. As appreciable in Figure 4a,b, after 1–3 h of incubation, many ethosomes were detectable in the stratum corneum, inside corneocytes, as well as in their interstices. Few ethosomes were also found inside keratinocytes, with a gradually dropping frequency from the upper to the basal strata (Figure 4c). Ethosome vesicles also occurred near cellular organelles such as mitochondria and smooth endoplasmic reticulum cisternae, as shown in Figure 4d. It is interesting to note that intact vesicles were present, suggesting that the structure of ethosomes was maintained through the skin strata. Remarkably, the TEM technique enabled unequivocally identifying nanovesicles, visualizing them directly inside the skin cells, avoiding markers that are otherwise needed in the case of fluorescence microscopy. The TEM strategy indeed prevents the risk of underestimating the signal given by the NP/nanovesicles in the case of autofluorescent tissues, such as the skin [151]. 

Ex vivo studies of NP–skin interaction were performed by fluorescent microscopy or by FACS. Nevertheless, this latest technique did not allow us to investigate the complete tissue architecture or to preserve the tissue structure, since it requires separation of cells from the tissue to obtain monodispersed cells in a liquid flow for single-cell analysis [152]. To overcome this limitation, confocal microscopy represented a more suitable technique for ex vivo and in vivo investigation of skin layers. Indeed, this non-invasive imaging technique resulted in a resolution close to the histological investigation [147].

Mahe et al. investigated the potential of utilizing confocal microscopy to elucidate the penetration and cellular uptake of solid polystyrene NPs and virus particles in murine skin as part of vaccine delivery strategies. By employing in vivo fibered-based confocal microscopy, alongside fluorescence microscopy on cryosections and cell separation techniques, the study aimed to understand the efficacy of transcutaneously applied particulate vaccine delivery routes. The research demonstrated that both 40 and 200 nm NPs, along with modified vaccinia Ankara expressing the green-fluorescent protein particles, were capable to penetrate deeply into hair follicles and to be internalized by perifollicular antigen-presenting cells [153].

The association of NPs with fluorophores can simplify their imaging detection, however, investigating fluorescence in skin tissues might be challenged due to its high autofluorescence. Indeed, skin proteins and pigments evidence their fluorescence, while melanin acts like a filter that could alter the interpretation of results [154]. A suggested method to better discriminate fluorescent NPs from the background is combining confocal imaging with spectrofluorimetric analysis [154].

**Figure 5:**
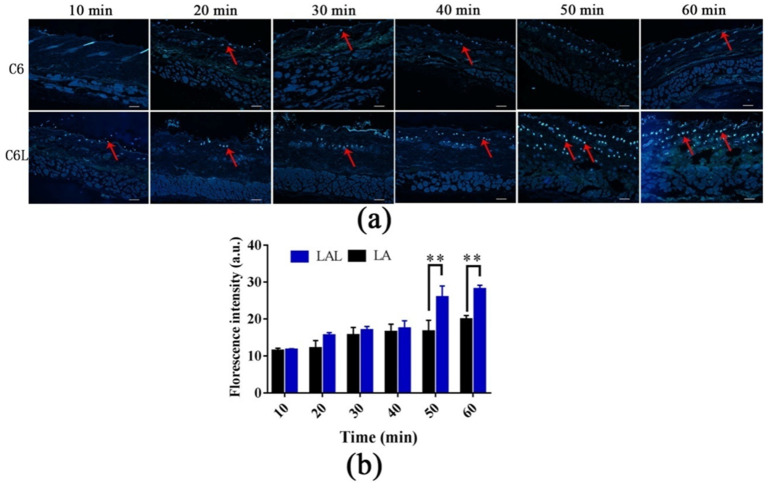
Analysis of licochalcone A (LA) or LA skin keratin liposomes loaded (LAL) distribution in abdominal skin of SD rats. (**a**) The fluorescence images of the skin samples treated with C6 (LA substitute) and C6L for 10, 20, 30, 40, 50, and 60 min. The scale bar is 100 μm; (**b**) the quantitative fluorescence intensities of C6 and C6L into skin at different time (** *p* < 0.01 vs. C6, *n* = 3) [139].

Wu et al. explored the distribution and penetration dynamics of Coumarin-6 (C6, as substitute of licochalcone A) and C6-loaded skin keratin liposomes (C6L) within the skin, shedding light on their potential as effective drug delivery systems. In this regard, Figure 5 depicts the fluorescence patterns observed over time. Initially, the fluorescence signal (indicated by red arrows) predominantly localized within the hair follicles, indicative of preferential accumulation in these structures. Nevertheless, after 1 h the fluorescence appeared more diffused across the skin, indicating the liposome penetration beyond the confines of the hair follicles and into the adjacent tissue. Additionally, in Figure 5b, the C6L exhibited deep penetration into the skin in a time-dependent manner. The fluorescence intensities of the C6L within the skin at 50 min and 60 min surpassed those of the free C6, indicating enhanced penetration and retention capabilities of the liposomal formulation [139].

Confocal microscopy can be coupled with Raman spectroscopy [3,160]. Ramzan et al. examined the human skin penetration of ketoconazole-loaded SLN ex-vivo using confocal Raman microscopy. They explored the efficacy of ketoconazole-loaded SLN to pass through the skin layers, revealing their ability to penetrate beyond the stratum corneum into the viable epidermis. Confocal Raman spectroscopy provided detailed insights into the NP penetration dynamics, showing the increased permeation capabilities of ketoconazole-loaded SLN compared to conventional formulations [161].

Synchrotron-based Fourier transform infrared microspectroscopy is a non-invasive technique for the molecular characterization of skin without labeling. Chemical maps identify the structure of lipids and proteins in the stratum corneum and epidermis and, based on spectra analysis, allowed us to identify the NP interaction with the skin strata [153]. Costa Lima and colleagues supported different structural changes in lipids and protein organization of the skin, induced by fucoidan/chitosan NPs with different molecular weights [158]. Even though this technique is one of a few methods for molecular investigation of skin structure changes, the use of synchrotron radiation makes it not easily accessible to many researchers.

Touloumesa et al. introduced HLM as a technique suitable for mapping the 2D distribution of TiO_2_ and ZnO NPs in the exposed dermal areas of cleared skin tissue sections [159]. Thanks to the pronounced optical scattering of TiO_2_ NPs, correlated hyperspectral microscopy was employed to accurately quantify their 2D distribution within the skin tissue section context [159]. Similarly, Mahdieh et al. demonstrated the potential of hyperspectral dark-field microscopy to investigate the release of silver NPs from fiber meshes into mouse skin tissue. This advanced technique offered a comprehensive understanding of the precise localization of silver NPs within the skin, overcoming conventional tissue staining methods [162].

The versatility of hyperspectral techniques extends beyond metal-based NPs, encompassing lipid NPs, polymers, peptides, and more. Once reference spectra were collected into spectral libraries, the samples could be analyzed using HLM to determine the location and composition of NPs accurately [149]. However, to the best of our knowledge, the utilization of HLM is still limited with regard to skin applications, indicating a gap in current research and suggesting an unmet need. Indeed, the technique is promising as a valuable tool for future investigations in this field, given its potential to provide detailed spectral information and spatial mapping.

Liu et al. employed enhanced dark-field hyperspectral imaging (EDF-HSI) for both in vitro and in vivo analyses of peptide NPs. These NPs were synthesized via the covalent self-assembly of dipeptides and genipin [163]. Remarkably, the optical scattering intensity of these peptide NPs was heightened through an amidation process. This strategy facilitated the observation and tracking of NPs within cellular and nematode environments using EDF-HSI. The findings indicate that peptide nanomaterials can be tailored to possess optical scattering properties suitable for EDF-HSI, enabling better investigation of the behaviors of biomaterials both in vitro and in vivo [163].

## 6. Conclusions

The possibility to deliver drugs through the skin is an interesting challenge in order to treat skin pathologies, as well as to prevent skin damage due to environmental factors. In this regard, notwithstanding encouraging results demonstrated the efficacy of many drug-loaded NPs, the use of different techniques is pivotal in order to better understand the influence of their physical-chemical features on skin interaction and to unequivocally detect their presence inside the skin. 

The present review has summarized the main techniques that can be employed nowadays to characterize NPs before application on skin tissues, as well as many research efforts to study NP interactions with the skin, by in vitro, ex vivo, and in vivo models. In the case of polymeric micelles, further investigation is needed to elucidate the precise mechanisms of action and optimize their efficacy in dermatological applications. The diverse properties and applications of inorganic NPs underscore their potential in various skin-related therapeutics, diagnostics, and cosmetics domains. However, comprehensive investigations are required to better elucidate their intrinsic structure and potential toxicological implications for efficacious and safe use in medical contexts. Dendrimers, particularly poly (amido amine) (PAMAM), PEGylated, and polyglycerol dendrimers, represent a promising avenue for innovative dermatological therapeutics. Despite the ongoing debate regarding their mechanisms of action and the extent of their enhancement in skin delivery, further studies are required to confirm dendrimer potential. Particularly microscopy techniques, associated or not to fluorescence, can give interesting information in this sense. 

Anyway, since the field is in continuous development, we believe that many researchers are now involved in these kinds of studies, thus the information can hardly be updated enough. Particularly, the HLM method might be one of the most useful techniques giving precious information both in the case of in vitro and ex vivo studies. Thus, it is likely that in the near future we will assist in a significant improvement and a better standardization of the characterization methods for NP detection in the skin. 

Due to the tremendous potential of the different techniques mentioned in this review, we strongly encourage researchers to associate as many methods as possible to improve the knowledge about the features of NP and their mechanisms of interaction with the skin.

## Figures and Tables

**Table 1:** Main types, characteristics, and applications of nanocarriers for skin administration.

NP	Solid Structure	Vesicle Structure	Mean Diameter, nm	Application	Ref.
Nanospheres	Yes	No	10–200	Skin care	[11,12]
Nanocapsules	Yes	No	5–1000	Prolonged antimicrobial	[13,14]
				Sunscreen	[15]
				Anti-inflammatory	[16,17]
Solid lipid nanoparticles	Yes	No	50–1000	Cosmetic use: sunscreens, anti-acne, anti-ageing actives	[18,19,20]
				Acne, psoriasis, ichthyosis	[21,22,23]
				Anti-inflammatory	[24,25]
Nanostructured lipid carriers	Yes	No	50–500	Anti-inflammatory (Immuno-suppressive)	[26]
				Local analgesic, anti-inflammatory	[27,28]
				Antimicrobial	[29]
				Anticancer	[30]
Nanoemulsions	Yes	No	10–1000	Cosmetic use: antioxidant, sunscreens, lipid carriers	[31,32,33]
				Nonsteroidal anti-inflammatory drug carriers	[34,35]
				Anticancer	[36,37]
				Antimicrobial	[38]
Liposomes	No	Yes	100–200	Antimicrobial treatment	[39,40]
				Eczema, psoriasis	[41,42]
				Anti-inflammatory	[43,44]
				Skin cancer	[45,46]
				Local anesthesia	[47,48]
Ethosomes	No	Yes	100–200	Skin pathology	[43,49]
				Skin cancer	[50,51]
				Skin infections	[52,53]
				Anti-inflammatory and analgesic	[54,55]
Micelles	No	No	20–300	Anti-inflammatory	[56,57]
				Antimicrobial	[58]
				Skin care	[59]
				Anticancer	[60]
Dendrimers	No	No	1–15	Skin care: anti-acne, sunscreen	[61,62]
				Antiviral (HSV)	[63,64]
				Anti-inflammatory	[65]
Inorganic NPs	Yes	No	3–6	Skin care: sunscreen	[66]
			20–150	Antimicrobial	[67]
			(coated)	Antioxidant	[68]
				Anticancer	[69]

**Table 2:** Investigation methods of NPs–skin interaction in vitro.

Method	Type of NP	Cell Model Type	Ref.
TEM	Ethosomes	Healthy human skin fibroblasts	[136]
	Ethosomes/Transethosomes	Immortalized keratinocytes, Human keratinocytes HaCaT cells	[73]
	Ethosomes/Transethosomes	HaCaT, Human primary fibroblasts	[137]
Flow cytometry	Silica NP	Immortalized keratinocytes, HaCaT cells and Human Skin Keratinocytes	[128]
	Silica NP	Different cell lines, among which HaCaT cells	[138]
Fluorescence	Ethosomes/Transethosomes	Human Keratinocytes and fibroblasts	[137]
microscopy	Skin keratin liposomes	Mouse skin melanoma, B16F10 cells	[139]
Confocal	Silica NP	Immortalized keratinocytes, HaCaT	[128]
microscopy	Zinc oxide NP	Immortalized keratinocytes, HaCaT	[140]
Raman	Silver NP	Mouse embryonic fibroblasts (NIH-3T3)	[141]
microscopy	Silver NP	Immortalized keratinocytes, HaCaT	[142]
Hyperspectral light microscopy	Metal oxide NP	Epithelial cell lines	[143]

**Table 3:** Methods of investigation NP–skin interaction ex vivo and in vivo.

Methods	Type of NP	Application	Ref.
TEM	Ethosomes	Reconstituted Human Epidermis RHE	[136]
	Ethosomes and transethosomes	Human skin explants	[151]
FACS	Polystyrene particles	Transcutaneous application on healthy human explants	[152]
Fluorescence microscopy	Polystyrene particles	Transcutaneous application on healthy human explants	[152]
Confocal microscopy	Skin keratin liposomes	Abdominal skin of Sprague Dawley rats	[139]
	Polystyrene particles (FluoreSpheres)	Mice skin cryosections	[153]
	Polymer NP	Rat skin explants	[154]
	Ethosomes	Bama mini-pigs explants	[155]
Fibered confocal fluorescence microscopy	Polystyrene particles	In vivo mice	[153]
Raman microscopy	Silver NP	Porcine ear skin explants	[156,157]
Syncrotron-based Fourier Transform Infrared Microspectroscopy	Polyelectrolyte complexed polymeric nanoparticle	Porcine ears	[158]
Hyperspectral light microscopy	TiO_2_, ZnO	Skin tissue	[159]

## Data Availability

Not applicable.
